# In Vitro Evaluation of the Inhibitory Activity of Thymoquinone in Combatting *Candida albicans* in Denture Stomatitis Prevention

**DOI:** 10.3390/ijerph14070743

**Published:** 2017-07-08

**Authors:** Ahmad M. Al-Thobity, Khalifa S. Al-Khalifa, Mohammed M. Gad, Mohammed Al-Hariri, Aiman A. Ali, Talal Alnassar

**Affiliations:** 1 Department of Substitutive Dental Sciences, College of Dentistry, University of Dammam, Dammam 32214, Saudi Arabia; mmjad@uod.edu.sa; 2 Department of Preventive Dental Sciences, College of Dentistry, University of Dammam, Dammam 32214, Saudi Arabia; kalkhalifa@uod.edu.sa; 3 Department of Physiology, College of Medicine, University of Dammam, Dammam 32214, Saudi Arabia; mtalhariri@uod.edu.sa; 4 Department of Biomedical Dental Sciences, College of Dentistry, University of Dammam, Dammam 32214, Saudi Arabia; Aiman.a.ali@gmail.com; 5 Department of Prosthetic Dental Sciences, College of Dentistry, Kind Saud University, Riyadh 11692, Saudi Arabia; dr_tmt@hotmail.com; 6 Department of Oral Rehabilitation, Dental College of Georgia, Augusta University, Augusta 30912, GA, USA

**Keywords:** *Candida albicans*, black seeds, denture base, denture stomatitis, thymoquinone

## Abstract

*Candida albicans* adhesion and proliferation on denture bases may lead to denture stomatitis, which is a common and recurrent problem in denture wearers. The goal of this study was to assess the inhibitory effect of thymoquinone incorporated in the polymethyl methacrylate denture base material against *Candida albicans*. Eighty acrylic resin specimens were fabricated and divided into eight groups (*n* = 10) according to thymoquinone concentrations of 0.5%, 1%, 1.5%, 2%, 2.5%, 3%, and 5% of acrylic powder. Two methods were used to evaluate the effect of thymoquinone on *Candida albicans*: the slide count and the serial dilution test. A multivariate analysis of variance (MANOVA) and the post-hoc Tukey’s Honestly Significant Difference (HSD) test were performed to compare the difference of means between the observations taken at various intervals with baseline. The *p* value was statistically significant at ≤0.05. According to the slide count and the serial dilution test, the mean number of adhered *Candida albicans* in the control group was 5436.9 ± 266 and 4691.4 ± 176.8; however, this number dramatically decreased to 0 ± 0 and 32.4 ± 1.7 in group 8 (concentration 5%). These results suggest that the incorporation of thymoquinone into the acrylic resin denture base material might be effective in preventing *Candida albicans* adhesion.

## 1. Introduction

Denture stomatitis (DS) is a highly prevalent disease in complete or partial denture wearers, which mainly affects the palatal mucosa [1,2]. Clinically, it appears as localized red erythematous patches or diffused patches beneath the denture [2,3,4]. Different factors may contribute to the development of DS, such as poor denture and oral hygiene, low flow of saliva, oral mucous membrane trauma, and microbial infection (basically *Candida* infection) [5,6,7,8,9]. DS has been found in approximately 30–75% of denture wearers and has a high rate of recurrence, even if treated with antifungal therapy [10,11,12,13].

The role of *Candida albicans* in DS has been investigated and strong evidence suggesting *Candida albicans* as the main fungal source has been shown [14,15,16]. The ability of *Candida albicans* to develop biofilms has been discerned as the key factor in the DS pathogenesis. However, the denture base material is porous in nature, which allows the *Candida albicans* to colonize and adhere into the denture surface. Furthermore, this biofilm formation and adhesion reduce the cleansing efficacy against the biofilms and increase its resistance to antifungal therapy [17,18,19,20].

Different management procedures have been developed to inhibit fungal growth into the denture base, such as denture cleansing modalities [21,22,23], the addition of antifungal medications into the denture lining or tissue conditioning materials [24,25], and the incorporation of antimicrobials into the denture base resin powder [26]. On one hand, the usage of denture cleansers may lead to deterioration of the denture base and increase its surface roughness, which makes it more susceptible to the biofilms’ accumulation [27,28,29,30]. On the other hand, studies have reported that the application of antimicrobial therapy into the denture materials could enhance the fungal resistance and reduce the medication effectiveness [19,20,31].

Nevertheless, the prevention of *Candida albicans* adherence to the denture base has been considered as an effective protocol in DS prevention [32,33,34,35]. Yodmongkol et al. [32] observed that when coating the acrylic resin specimens with silane-SiO_2_ nanocomposite films the adhesion of *Candida albicans* to specimens’ surfaces was reduced without affecting its physical properties.

Natural products have been investigated experimentally by mixing them with the denture base material to evaluate their effectiveness in the inhibition of *Candida albicans* growth [36,37]. Recently, Nawasrah et al. [36] reported that adding 1% of henna powder to the denture material resulted in a significant reduction in the *Candida albicans* count.

*Nigella sativa* is an annually flowering medicinal plant native to South and Southwest Asia; its seeds are commonly known as black seeds or black cumin [38]. *N. sativa* seed extract includes essential oil, alkaloids, fixed oil, proteins, and saponins. Its extract has been explored in the medical field and has been found to have antibacterial, anti-inflammatory, anti-oxidant, and antitumor properties [39,40,41,42]. Thymoquinone (TQ) is the major ingredient in *N. sativa* seed essential oil, which has been proved to have broad medical benefits [43,44,45]. In addition, *N. sativa* seed extract has been tested recently in the dental field for a potential therapeutic effect against dental caries [46], pulpal diseases [47], gingival and periodontal diseases [48], and oral ulcerations [49].

The lowest therapeutic concentration of a medical agent that inhibits the development and growth of any microorganism is known as the Minimum Inhibitory Concentration (MIC) [50]. Harzallah et al. [51] reported that 2.13 mg/mL (MIC) of TQ has a strong antibacterial action against *Streptococcus mitis* and *Streptococcus mutans* as cariogenic strains. The therapeutic effect of TQ in the MIC against *Candida albicans* for the prevention of DS has not been reported in the literature. The goal of this study was to assess the MIC of TQ incorporated in the polymethyl methacrylate (PMMA) denture base material against *Candida albicans*.

## 2. Materials and Methods

The sample size was calculated using the results of a previous study [36]. The sample size was calculated to be 20 per group keeping a confidence interval of 95% and a power of at least 80%. A total of 80 specimens of heat polymerized acrylic resin (Major base 20 resin; Prodotti Dentari SPA, Moncalieri, Italy) using a negative metal mold with the dimensions of 10 × 10 × 3 mm was prepared and waxed. Wax specimens were flasked in stone then wax burned-out to create mold space. Acrylic resin material was prepared by the adding of TQ (thymoquinone ≥98%; Sigma-Aldrich, Taufkirchen, Germany) in concentrations of 0.5, 1, 1.5, 2, 2.5, 3 and 5 wt % of acrylic powder and properly mixed to attain a homogenous color. To fabricate acrylic resin specimens, polymer and monomer were measured and mixed according to manufacturer instructions. Mixing was done in a porcelain jar, which was kneaded by hand upon achieving a dough-like consistency to increase its homogeneity and integrity. At the dough stage, the mixture was packed and then processed in a heat curing unit at 74 °C for 2 h and 100 °C for 1 h. After the curing of all the specimens, the flasks were brought down to room temperature and deflasked. The excess resins of the deflasked specimens were removed, and then the specimens were finished and then stored at 37 °C for 24 h in sterile distilled water to remove any residual monomers. According to the different TQ concentrations, the specimens were divided into 8 groups (*n* = 10) represented in Table 1.

### 2.1. Microbiology Test

#### 2.1.1. Exposing Acrylic Specimens to *Candida albicans*

Before microbiologic valuation, the acrylic specimens were sterilized in an autoclave (Ritter M11 UltraClave; Midmark International, Versailles, OH, USA) for 15 min under 15 bar at 121 °C. All acrylic plates’ specimens were immersed in artificial saliva (A.S. Orthana, Biofac A/S, Kastrup, Denmark) containing 2,000,000 cells of *Candida albicans* (ATCC 10231) for two weeks at a temperature of 37 °C (Table 2). The acrylic plates were washed three times with phosphate-buffered saline (PBS) to remove non-adherent cells and then placed in sterile tubes with 1 mL of Sabouraud’s dextrose broth (SDB Acumedica Co., Manufacturers, Inc., Lansing, MI, USA) for 2 days. The plates were then vibrated using a vortex mixer for 10 min followed by centrifuging the tubes at 4500 rpm for 5 min to get the concentrated bullet of *Candida albicans*. At this stage, two methods were used to count the number of alive *Candida albicans* for each sample:

#### 2.1.2. Evaluation

After centrifuging, the acrylic resin plates were removed from their tubes, and the concentrated pellet was collected from the tube. Two methods of evaluation were used to calculate the amount of *Candida albicans* adhered to each acrylic resin specimen as follows:

##### Slide Count

Samples were placed on a special slide count (Neubauer Slide Counter; Chambers-Marienfeld, Lauda-Konigshofen, Germany) after adding 2.5 µL of Trypan Blue 0.4% solution in phosphate (MP-Biomedicals, Santa Ana, CA, USA) to 7.5 µL of each sample to be evaluated under light microscope. Trypan Blue stain can differentiate between dead and alive *Candida albicans* cells; dead *Candida albicans* usually appear blue while alive *Candida albicans* appear transparent with a blue peripheral line. To count the number of *Candida albicans*, a light microscope with a magnification of 10× was used. *Candida albicans* were counted in two squares out of the four main squares of the slide count and multiplied by 2 to find out the total number of *Candida albicans* in each slide.

##### Serial Dilution Test

**Figure 1:**
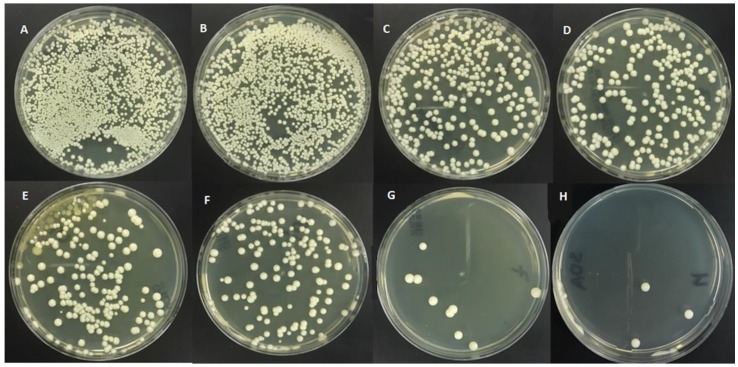
Cultures of *Candida albicans* colonies based on different concentrations of thymoquinone: (**A**) Control group (0%); (**B**) 0.5%; (**C**) 1%; (**D**) 1.5%; (**E**) 2%; (**F**) 2.5%; (**G**) 3%; (**H**) 5%.

A 10 µL of each bullet was taken, and then it was diluted serially and spread on a petri dish containing Sabouraud dextrose agar (SDA) (Acumedica Co., Manufacturers, Inc.) and incubated for 48 h at 37 °C. A marker pen counter (Colony Counter; Bel-Art Scienceware, Wayne, NJ, USA) was used to count the number of *Candida albicans* colonies in each quadrant where acceptable growth was noted and the final number was corrected for the dilution factor. If the number of colonies was 500 or more, it was considered as an overgrowth (Figure 1) [37].

### 2.2. Statistical Analysis

SPSS-20.0 (IBM Inc., Armonk, NY, USA) was used for the statistical data analysis. The results of the *Candida albicans* count from the two different methods were formulated into arithmetic means and standard deviations. The multivariate analysis of variance (MANOVA) was applied to compare the mean effect on each interval with the baseline. The post-hoc Tukey’s Honestly Significant Difference (HSD) test was performed to compare the difference of means between the observations taken at various intervals with baseline. If the *p* value was ≤0.05, then it was considered statistically significant.

## 3. Results

The antifungal effect of TQ on *Candida albicans* was examined using different concentrations. The mean and standard deviation values were obtained for each group. It can be observed, that the use of TQ at a concentration starting from 0.5% was associated with a significant reduction of *Candida albicans* in the slide count (Table 3). In addition, the inhibitory effect of TQ on *Candida albicans* increased significantly with the concentration of TQ. Interestingly, at the concentration 3% and above, there were no signs of any fungal growth using the slide count.

To ensure the antifungal effect of TQ, colonies of *Candida albicans* were counted after applying the same doses of TQ using cell culture counts (Figure 1). Table 3 shows the significant effect of TQ has on live *Candida albicans* using concentrations of 0.5% and higher. There were no differences in the antifungal effect of TQ in either methods, it can be seen that the significant effect of TQ, which indicates the significant inhibitory effect of TQ on *Candida albicans*. However, the significant effect of TQ at a concentration of 2.5% and higher on *Candida albicans* was greater compared to the other concentrations.

## 4. Discussion

The goal of the present study was to assess the inhibitory effect of TQ (the active ingredient of *N. sativa*) as natural and safe compound on *Candida albicans* adherence to PMMA acrylic resins as an alternative method for the prevention of DS, which frequently occurs in patients who wear complete dentures [52]. Using the slide method and the serial dilution test, the results showed that the TQ at the MIC significantly reduced the number of *Candida albicans*. Furthermore, these results found that the adding of 0.5% of TQ to the PMMA led to a significant reduction of *Candida albicans*. By increasing the percentage of TQ from 0.5% to 5%, the number of *Candida albicans* dramatically decreased to zero using the slide count evaluation method.

Several studies have investigated the medicinal effect of the *N. sativa* extract in different dental applications [46,47,48,49]. Omar et al. [47] evaluated *N. sativa* oil as pulp capping medicaments in pediatric dentistry using the animal model. They found that there was less infiltration of the inflammatory cells and fewer degenerative changes after the application of *N. sativa* oil, when compared to the formocresol medicament. They concluded that the *N. sativa* had an anti-inflammatory effect and could be used as a pulp capping agent. Another study evaluated the effect of the biodegradable periodontal chip, including the use of TQ in the management of patients with chronic periodontitis. They found a significant reduction in the periodontal pockets, bleeding on probing and the plaque index, and a significant increase in the clinical attachment in subjects treated with TQ [48].

DS is a disease that is most commonly associated with the use of acrylic dentures. Many factors may contribute to the development of the denture stomatitis etiology; however, all of these factors are related to the ability of *Candida albicans* to adhere to and colonize on the dentures and oral mucosal surfaces [7]. Many studies have reported that mechanical and chemical cleansing for the removable prostheses are not adequate enough to eliminate contaminating microorganisms [27,28,29,30]. The increased antimicrobial resistance emphasizes the need for a study and evaluation of a new antifungal agent [17,18,19,20,53]. Different mechanisms have been suggested for the development of resistance to treatment regimes in *Candida albicans* [19,20,31,54]. A number of factors, such as nutritional factors, radiotherapy, surgical procedures, poor oral hygiene, and others, have been associated with the increasing frequency of *Candida albicans* intra-orally [55].

Several studies have addressed ways to decrease the formation and the development of adherent biofilms through modifying the denture base, modifying the denture surface, or through chemical modification [21,22,23,24,25]. Modifying the denture base materials by incorporating TQ was investigated in this study in an attempt to control and prevent the adhesion of *Candida albicans* on the surface of acrylic resin dentures.

The *Nigella sativa* extract (6.6 mL/kg daily for the 3 days) has shown a marked ability to inhibit the growth of *Candida albicans* in infected mice [56]. Few studies have been conducted on the effect of using TQ on *Candida albicans.* One study has assessed the in vitro inhibitory activity of the ethanol extracts of *Nigella sativa*, along with those of five other plants, against the oral candidal isolates collected from 175 patients. It has been recorded that the smallest inhibition zone was noticed with a 100 μg/mL concentration of *Nigella sativa* [57]. Another study compared in vitro the antifungal activity of nanoparticulate TQ versus microstructured TQ, ketoconazole and amphotericin B against *Candida albicans*. The study found nanosized TQ to be 2 to 4 times more active against *Candida* yeasts and *Candida* biofilm [58].

The present work was conducted to evaluate the effect of TQ, as natural product, against *Candida albicans* to prevent or treat DS. Based on our results, TQ exhibited antifungal effect at a 0.5% concentration in the PMMA denture base material. These results also show that TQ can significantly inhibit the growth of *Candida albicans* in agreement with a previous study that found TQ exhibited a potent growth-inhibitory effect against *Candida albicans* [55]. The results also suggest that the concentrations of TQ (2.5%, 3%, and 5%) are effective for inhibiting *Candida albicans.*

The ability of TQ to inhibit the *Candida albicans* as observed by the present study will promote further investigations in determining the usefulness of TQ in combatting *Candida albicans.* Additionally, this study may promote the use of TQ as an effective alternative method to prevent and/or treat patients with this pathogen. The limitations of this study were that the oral environment consisted of several microorganisms, not only *Candida albicans*, and that no aging procedure was performed. In the future studies, under better-simulated conditions, varied microorganisms, including biofilm formations, should be evaluated. However, investigating the biocompatibility of PMMA/TQ composite, comparing the effect of TQ with different antifungal effect on Candida adhesion and evaluating the effect of the TQ addition on the physical properties of the acrylic resin denture base materials are necessary.

## 5. Conclusions

Within the limitations of this study, it could be concluded that the incorporation of TQ as a natural compound into the acrylic resin denture base material could be effective in preventing *Candida albicans* adhesion and proliferation on the denture surface. Further investigations on the physical properties of the PMMA/TQ composite are required.

## Figures and Tables

**Table 1:** Tested groups and description according to thymoquinone (TQ) concentrations.

Groups	Description
0% (Control)	heat polymerized specimens
0.5%	heat polymerized specimens incorporated with 0.5% TQ
1%	heat polymerized specimens incorporated with 1% TQ
1.5%	heat polymerized specimens incorporated with 1.5% TQ
2%	heat polymerized specimens incorporated with 2% TQ
2.5%	heat polymerized specimens incorporated with 2.5% TQ
3%	heat polymerized specimens incorporated with 3% TQ
5%	heat polymerized specimens incorporated with 5% TQ

**Table 2:** Composition of artificial saliva.

Artificial Saliva	Composition
A.S. Orthana, Biofac A/S, Kastrup, Denmark	Mucin, methyl-4-hydroxybenzoate, benzalconium chloride, ethylenediaminetetraacetic acid (EDTA), H_2_O_2_, xylitol, peppermint oil, spearmint oil and mineral salts

**Table 3:** Effect of different concentration of TQ on *Candida albicans* count.

TQ Concentration	Slide Count	Serial Dilution Test
	Mean ± SD	Mean ± SD
0% Control	5436.9 ± 266	4691.4 ± 176.8
0.5%	3776.10 ± 98.8	3334.7 ± 121.2
1%	3037.4 *** ± 39.2	2619.4 *** ± 50.1
1.5%	980.2 ** ± 10.8	894.6 ** ± 32.3
2%	466 ** ± 6.5	310.3 ** ± 8.2
2.5%	166.5 * ± 6	91.9 * ± 4.5
3%	0 * ± 0	53.9 * ± 2.0
5%	0 * ± 0	32.4 * ± 1.7

^a^ Significantly different from control group; *** Significantly different at 0.05; ** Significantly different at 1; * Significantly different at 1.5; using one-way analysis of variance at *p* = 0.05. SD: Standard Deviation.
